# Multimodality PET and Near-Infrared Fluorescence Intraoperative Imaging of CEA-Positive Colorectal Cancer

**DOI:** 10.1007/s11307-023-01831-8

**Published:** 2023-06-21

**Authors:** Thinzar M. Lwin, Megan Minnix, Lin Li, Anakim Sherman, Teresa Hong, Jeffery Y. C. Wong, Tove Olafsen, Erasmus Poku, Michael Bouvet, Yuman Fong, John E. Shively, Paul J. Yazaki

**Affiliations:** 1 Department of Surgery, City of Hope National Medical Center, Duarte, CA USA; 2 Department of Immunology & Theranostics, Beckman Research Institute, City of Hope, 1500 Duarte Road, Duarte, CA 91010 USA; 3 Department of Radiation Oncology, City of Hope National Medical Center, Duarte, CA 91010 USA; 4 Small Animal Imaging Core, Beckman Research Institute, City of Hope, Duarte, CA 91010 USA; 5 Radiopharmacy, Beckman Research Institute, City of Hope, CA 91010 Duarte, USA; 6 Department of Surgery, University of California, San Diego, La Jolla, CA USA; 7 VA San Diego Healthcare System, La Jolla, CA USA

**Keywords:** Multimodality imaging, Anti-CEA, Antibody-dye conjugate

## Abstract

**Purpose:**

Molecular imaging is a major diagnostic component for cancer management, enabling detection, staging of disease, targeting therapy, and monitoring the therapeutic response. The coordination of multimodality imaging techniques further enhances tumor localization. The development of a single agent for real-time non-invasive targeted positron emission tomography (PET) imaging and fluorescence guided surgery (FGS) will provide the next generation tool in the surgical management of cancer.

**Procedures:**

The humanized anti-CEA M5A-IR800 “sidewinder” (M5A-IR800-SW) antibody-dye conjugate was designed with a NIR 800 nm dye incorporated into a PEGylated linker and conjugated with the metal chelate p-SCN-Bn-deferoxamine (DFO) for zirconium-89 PET imaging (^89^Zr, half-life 78.4 h). The dual-labeled ^89^Zr-DFO-M5A-SW-IR800 was evaluated for near infrared (NIR) fluorescence imaging, PET/MRI imaging, terminal tissue biodistribution, and blood clearance in a human colorectal cancer LS174T xenograft mouse model.

**Results:**

The ^89^Zr-DFO-M5A-SW-IR800 NIR fluorescence imaging showed high tumor targeting with normal liver uptake. Serial PET/MRI imaging was performed at 24 h, 48 h, and 72 h and showed tumor localization visible at 24 h that persisted throughout the experiment. However, the PET scans showed higher activity for the liver than the tumor, compared to the NIR fluorescence imaging. This difference is an important finding as it quantifies the expected difference due to the sensitivity and depth of penetration between the 2 modalities.

**Conclusions:**

This study demonstrates the potential of a pegylated anti-CEA M5A-IR800-Sidewinder for NIR fluorescence/PET/MR multimodality imaging for intraoperative fluorescence guided surgery.

**Supplementary Information:**

The online version contains supplementary material available at 10.1007/s11307-023-01831-8.

## Introduction

Multi-modality diagnostic imaging is a major component of cancer care enabling real-time non-invasive detection, staging of disease, targeting therapies, and monitoring for a therapeutic response [1]. The development of anti-tumor antibody-based multimodality imaging probe that integrates both PET and NIR fluorescence provides the potential for highly synergistic properties for patient disease staging, optical and radionuclide based surgical resection and monitoring of therapeutic responses in oncology [2-7]. Positron emission tomography (PET) imaging in combination with CT or MRI provides non-invasive, quantitative, high-resolution images of a radiotracer probe for tumor detection based on metabolism or antigen expression, along with anatomic registration [8, 9]. Real-time intraoperative near infrared (NIR) fluorescence imaging provides in situ visual information that complements anatomic information. Optical imaging technology has been used to perform fluorescence guided surgery (FGS) and has demonstrated an impact on the outcome of cancer surgery for the detection of small lesions, surgical margins, and positive lymph nodes in preclinical and early-stage clinical trials [10-14]. Optical imaging provides in situ visual information that complements anatomic information. However, optical imaging technology is limited by low energy photons which are scattered within deep tissues, limiting this technology primarily to superficial lesions [15]. This inherent limitation can be overcome with dual labeling agents as radionuclide probes can provide quantitative and deep tissue penetration but do not provide spatial correlation [5].

The humanized anti-CEA hT84.66-M5A monoclonal antibody (M5A mAb) as a radiolabeled theranostic agent is demonstrating CEA-positive tumor specificity in ongoing first-in-human PET imaging trials for colorectal cancer (NCT02293954 and NCT05245786). Supported by this clinical evidence, we have developed the M5A mAb conjugated with a NIR 800 nm dye for fluorescence image guided surgery. The M5A-IR800 was able to visualize CEA-positive primary and metastatic disease in colorectal and pancreatic cancer xenograft mouse models [16-18]. However due to the hydrophobicity of the IR800 dye, higher dye/mAb ratios resulted in faster blood clearance. The current M5A-IR800 “sidewinder” (SW) incorporates a long-chain monodispersed polyethylene glycol (PEG) linker with an internal lysine sidechain for IR800 dye attachment that demonstrated high dye-to-mAb ratios for enhanced signal detection while retaining similar *in vivo* blood clearance properties to the parental M5A mAb [19]. These studies showed the M5A-SW-IR800 optimal tumor-to-background ratio occurred 48–96 h post-injection. We chose the PET radionuclide zirconium-89 (Zr-89) due to its half-life of 78.4 h, making it an ideal match for this PET-NIR fluorescence imaging study. In this study, the anti-CEA M5A mAb was modified by click chemistry to link the azido-SW-IR800 dye into the IGg1 hinge domain for ease of manufacture, followed by orthogonal conjugation with the metal chelate p-SCN-Bn-deferoxamine (DFO). We evaluated the ^89^Zr-DFO-M5A-SW-IR800 as a multimodality imaging agent in a colorectal cancer xenograft mouse model.

## Materials

The antibody anti-CEA M5A has been previously described (20). dPEG sidewinder NH_2_-dPEG_12_-Lys(t-boc)-NH-m-dPEG_24_ was obtained from Quanta Biodesign, Plain City, OH (product number 11598). Azido-PEG_4_-active ester (product number BP-20518) and DIBCO-amine (product number BP-22066) were obtained from BroadPharm, San Diego, CA. Bromoacetamido-DBCO was synthesized as previously described (Kujawski et al., 2019). Tris(2-carboxyethyl) phosphine hydrochloride (TCEP, product number C4706) was obtained from Sigma-Aldrich (St. Louis, MO). p-SCN-Bn-deferoxamine (DFO, product number B-705) was obtained from Macrocylics, Dallas, TX.

## Methods

### Synthesis of Anti-CEA DFO-M5A-IR800 Sidewinder

The humanized anti-CEA hT84.66-M5A (M5A) monoclonal antibody (mAb) [20] was conjugated with a linear defined polyethylene glycol (dPEG)-IR800 sidewinder (SW) into the cysteines of the IgG1 hinge using synthesis and click chemistry methods previously described [19, 21]. Briefly, Supplemental Scheme 1 shows the dPEG sidewinder NH_2_-dPEG_12_-Lys(t-boc)-NH-m-dPEG_24_ (Compound 1, 23.8 mg, 12.4 nmol) was reacted with azido-PEG4-NHS (15.0 mg, 38.6 nmol) at the molar ratio of 1:3.1 in water at pH 7–7.5 overnight under argon. Compound 2 was purified using reverse phase on a Gemini C18 HPLC column (Phenomenex, Torrance, CA) and the molecular mass verified by ESI/MS on a Thermo Finnigan LTQ mass spectrometer ([M + H] + ^1^: cal 2189.63, obs: [M + H]^+1^: 2189.33). The azido-SW-IR800 was obtained by removing the tert-butyloxycarbonyl (t-boc) protecting group and reacting with the non-aryl sulfonated IR800 nm dye (excitation 780 nm emission 815 nm) to form Compound 3 [22]. The M5A mAb (2 mg, 13.33 nmol) was reduced with 30 molar excess of tris (2-carboxyethyl) phosphine (TCEP) at 37 °C for 2 h under argon. TCEP was removed by using a desalting spin column (Zeba, 7KDa MW cutoff, Thermo Scientific, Waltham, MA). The reduced M5A was reacted with 20-fold molar excess bromoacetamido-DBCO at RT overnight under argon. The purified M5A-DBCO was dialyzed in PBS to remove unconjugated DBCO (2L × 5). The M5A-DBCO (2 mg, 13.33 nmol) was mixed at a 1:10 molar ratio with azido-SW-IR800 (0.4 mg, 133.33 nmol) at RT overnight. The mixture was purified by using a HPLC size exclusion chromatography (SEC, Superdex-200, Cytiva, Waltham, MA) to produce M5A-PEG4-CO-NH_2_-dPEG_12_-Lys-(NH-IR800)-NH-m-dPEG_24_ (M5A-SW-IR800). The M5A-SW-IR800 (1.5 mg, 10 nmol) was conjugated with p-SCN-BN-deferoxamine (DFO, 76 µg, 100 nmol) at a conjugation ratio of 1:10, dialyzed vs. PBS to produce DFO-M5A-SW-IR800. Antibody conjugates analyzed by Agilent 6520 QTOF mass spectrometry as previously described[21], gave an estimate of 4–5 DFOs per mAb (Supplemental Figure S1).

### Radiolabeling

The DFO-M5A-SW-IR800 was radiolabeled with Zr-89 (3D Imaging, Little Rock, AR, specific activity 14.1 µCi/µg, in 1 M HEPES for 1 h at 43 °C) The radiolabeling efficiency was 98% by instant thin-layer chromatography. The ^89^Zr-DFO-M5A-SW-IR800 was purified by HPLC SEC with a single peak that corresponded to a molecular weight slightly larger than IgG1 as expected for the pegylated mAb. The final product was formulated by mixing “hot” ^89^Zr-DFO-M5A-SW-IR800 with unlabeled DFO-M5A-SW-IR800 to a final dose of 20 µCi/50 µg/mouse. Incubation with soluble CEA (20 molar excess) showed > 95% immunoreactivity by an *in vitro* molecular weight shift assay and stability study showed the product was stable at least to 72 h by HPLC SEC [23].

### Animal Studies

The human colon carcinoma cell line LS174T was obtained from and validated by ATCC (Manassas, VA), cultured at 37 °C with 5% CO_2_ in ATCC-recommended media with 10–20% heat inactivated fetal bovine serum (FBS, Corning, Corning, NY) supplemented with 5 mM L-glutamine (L-glut, MP Biomedicals LLC). LS174T was grown in DMEM, 10% FBS, and L-glut. Animal studies were performed in accordance with protocols 19,052 and 91,037 approved by the City of Hope Institutional Animal Care and Use Committee, in accordance with the National Institute of Health Office of Laboratory Animal Welfare guidelines. Seven- to 8-week-old female athymic mice (Charles River Laboratories, Wilmington, MA) were injected subcutaneously in the flank region with 10^6^ LS174T human colon carcinoma cells. After 12 days, when tumor masses were in the range of 100–300 mg, 8 mice were injected with 200 µl immunoglobulin (IVIG, 5 mg/ml) 2 h prior to intravenous injection of the ^89^Zr-DFO-M5A-SW-IR800 (20 µCi/50 µg). Mice were randomly grouped into 2 cohorts: 4 mice for PET/MR/NIR fluorescence imaging and 4 mice for blood clearance. The PET/MR imaging was performed on a pre-clinical 7 T PET/MR (MR Solutions, Guildford, Surrey, UK) at 24 h, 48 h, and 72 h post-injection. Shortly before scanning, mice were anesthetized with isoflurane and placed in a prone position on a heated imaging bed with respiratory monitoring. Static PET scans were acquired for 10 min at 24 h and 48 h and 15 min at 72 h. Each PET scan was followed by T1w and T2w whole body MRI for anatomical localization. PET data were reconstructed and co-registered with MR images using A Medical Imaging Data Examiner (AMIDE) [24]. Tumor to liver ratios were determined by drawing ellipsoid regions of interest (ROIs) of 3 × 3 × 3 mm in the tumors and 5 × 5 × 5 mm in liver. After the last PET/MR scan was acquired, the mice were euthanized, transferred to a Pearl Trilogy small animal imaging station (Li-Cor Bioscience, Lincoln, NB) for white-light and NIR 800 nm channel laser source imaging at 3 stages: non-invasive, open flap, and dissected organs using the manufacturer’s settings. For the other 4 mice, ^89^Zr-DFO-M5A-SW-IR800 blood clearance was measured by microcapillary sampling of 10 µl of blood from the tail vein at 0, 2, 4, 24, 48, and 72 h post-injection and counted using calibrated PerkinElmer gamma counter. After the last blood sample or image was acquired, all animals were euthanized, necropsy performed and organs weighed (tumor, blood, heart, lung, liver, stomach, small and large intestine, spleen, kidneys, right quadricep muscle, carcass) and counted for radioactivity. All data are mean values and have been corrected for radioactive decay back to the time of injection, allowing organ uptake to be reported as percent of the injected dose per gram (% ID g^–1^) with standard errors. All statistical analyses were conducted using Prism version 9 (GraphPad Software, San Diego, CA).

## Results

**Fig. 1:**
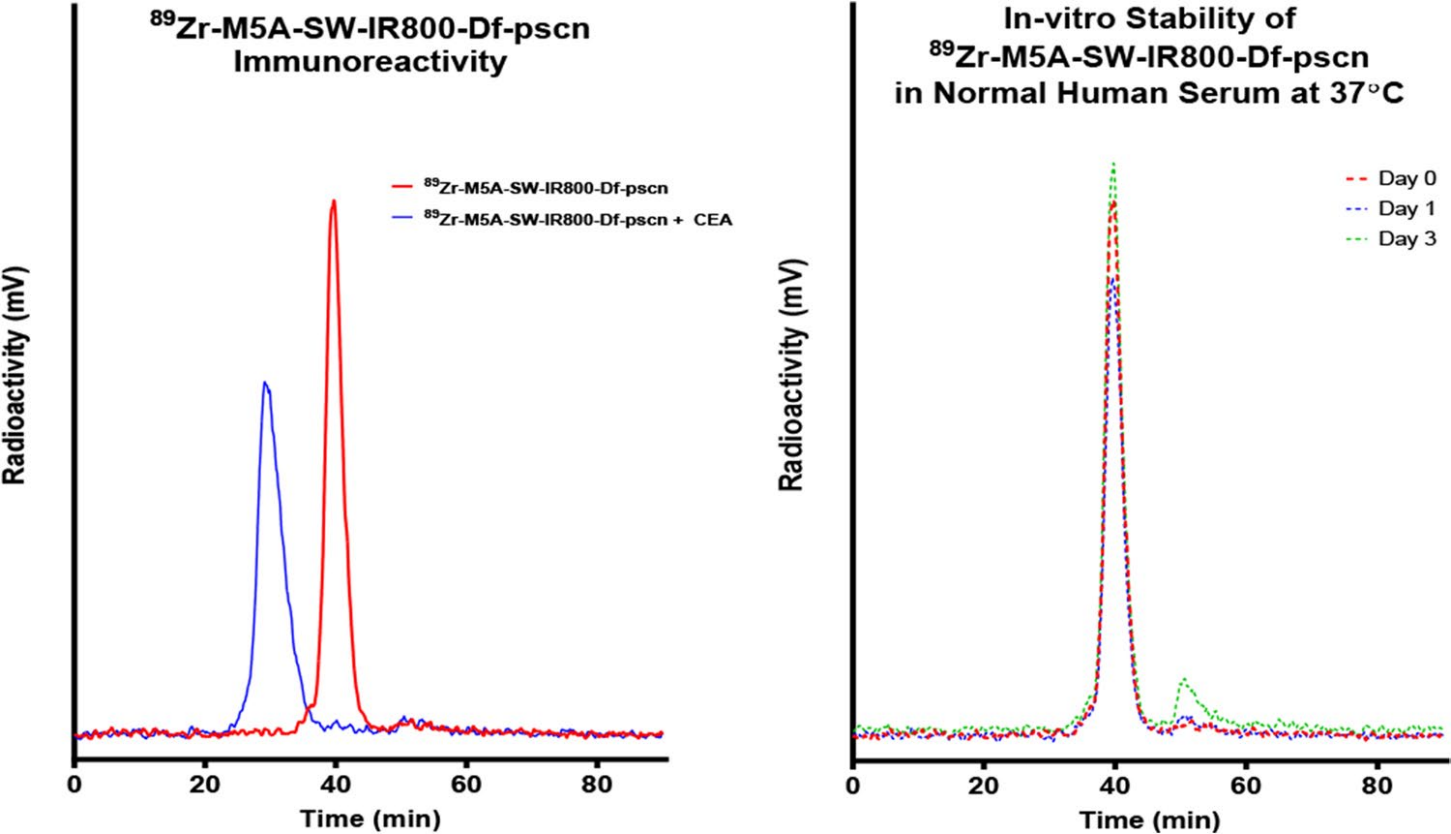
^89^Zr-DFO-M5A-SW-IR800 immunoreactivity and *in vitro* stability. ^89^Zr-DFO-M5A-SW-IR800 was tested for immunoreactivity to soluble CEA (left panel) and *in vitro* stability in human serum for 72 h (right panel) by HPLC SEC

We developed a multimodality imaging probe based on the anti-CEA hT84.88-M5A monoclonal antibody (M5A mAb) for PET and NIR fluorescence intraoperative imaging of CEA positive cancers. We chose to use the positron-emitting radionuclide Zr-89 (half-life 3.3 days, E_β+,ave_ = 396 keV) to allow PET imaging to correspond with the optimal NIR fluorescence imaging time. The humanized M5A mAb was conjugated in the IgG1 hinge cysteines with a dPEG-sidewinder-IR800 dye by click chemistry and amine conjugation with p-SCN-BN-DFO. The anti-CEA DFO-M5A-SW-IR800 was radiolabeled with ^89^Zr, purified as a single peak (Fig. 1 Left panel, red line), shown to be 100% immunoreactive when incubated with soluble CEA (Fig. 1 Right panel, blue line) and stable in human serum for 72 h *in vitro* as determined by SEC (Fig. 1B).

The ^89^Zr-DFO-M5A-SW-IR800 was iv administered at a dose of 20 µCi/50 µg dose into 8 athymic mice bearing 10-day-old human colorectal cancer LS174T subcutaneous tumors. Four mice underwent imaging and 4 mice used for blood clearance studies [25].

**Fig. 2:**
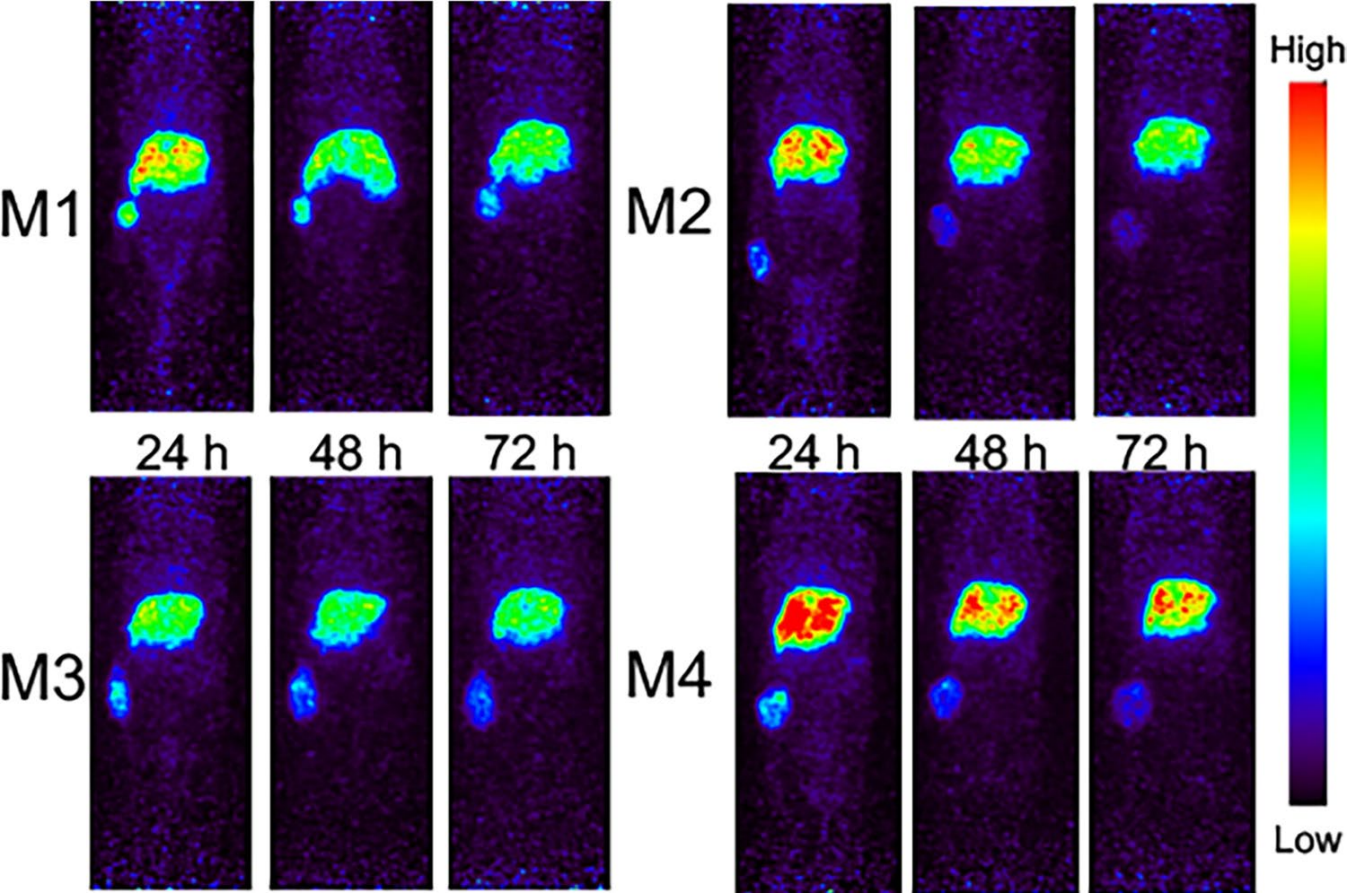
Serial immunoPET imaging. **A**.^89^Zr-DFO-M5A-SW-IR800 PET maximum intensity projection (MIP) coronal view scans are shown at 24 h, 48 h, and 72 h for all 4 mice (M1-4). **B** After the last images were acquired, organs were dissected, weighed and radioactivity counted, and data expressed as percent injected dose per gram (% ID/g)

Due to the surgery required for intra-vital NIR fluorescence imaging, the noninvasive PET/MRI imaging was performed in advance of the optical imaging. Serial real-time ^89^Zr-DFO-M5A-SW-IR800 PET/MR imaging was done using a MR Solutions PET/MR 7.0 T scanner. The PET maximum intensity projection (MIP) coronal view scans for all 4 mice are shown at 24 h, 48 h, and 72 h (Fig. 2). The ^89^Zr-DFO-M5A-SW-IR800 optical imaging demonstrate highly specific targeting of the human colorectal cancer subcutaneous tumor. The liver and tumor were the major tissues visible at all time points. While there were differences in signal intensity between the mice, all the PET MIP images show higher activity in the liver compared to the tumor.

**Fig. 3:**
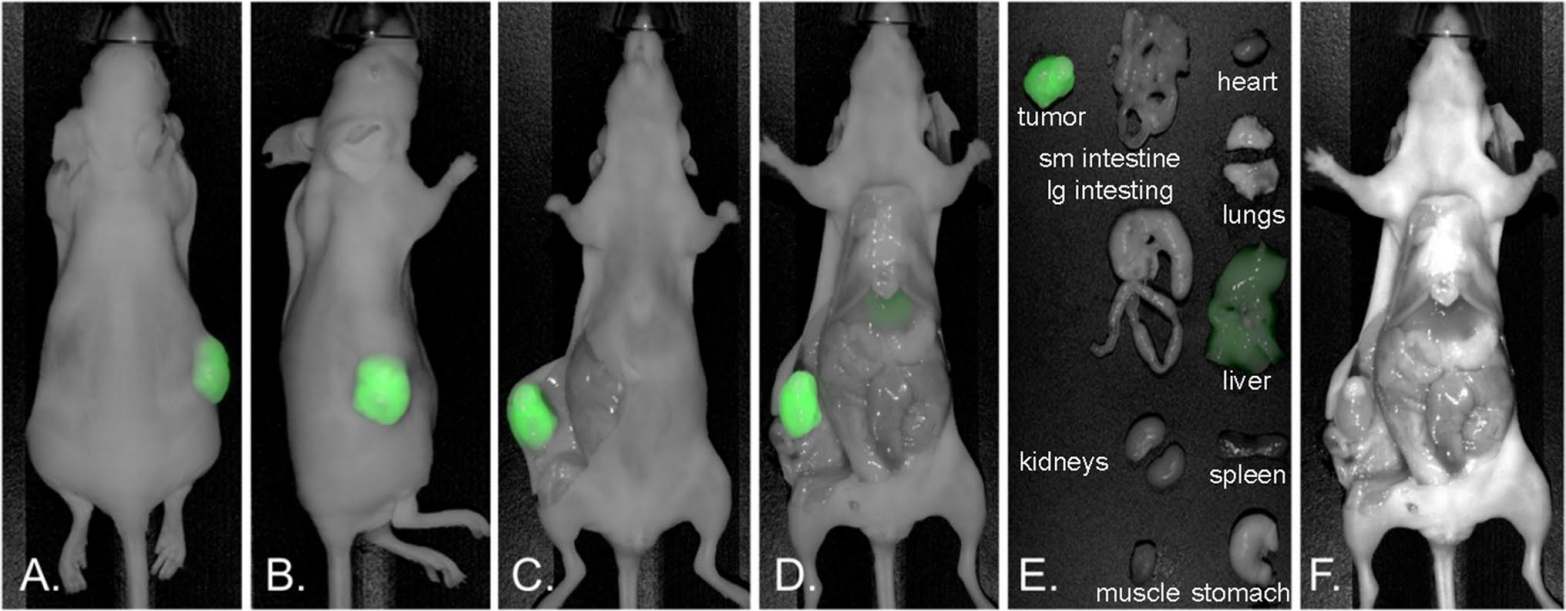
NIR fluorescence imaging. The ^89^Zr-DFO-M5A-SW-IR800 was administered into athymic mice bearing human colorectal cancer LS174T subcutaneous tumors. NIR fluorescence images were acquired from following orientations: **A** non-invasive dorsal view, **B** right flank view, **C** ventral open skin flap, **D** open intraperitoneal cavity, **E** dissected organs, and **F** bright white image of open intraperitoneal cavity at 72 h for a representative mouse. Organs were dissected, weighed and fluorescence intensity measured, data expressed as fluorescence intensity per gram

NIR fluorescence imaging was performed at 72 h using a Pearl Trilogy Small Animal Imaging System. Figure 3 shows NIR fluorescence images acquired at the following positions: (A) non-invasive dorsal view, (B) right flank view, (C) ventral open skin flap, (D) open intraperitoneal cavity, (E) dissected organs, and (F) bright white light image of the open intraperitoneal cavity from a representative mouse.

**Fig. 4:**
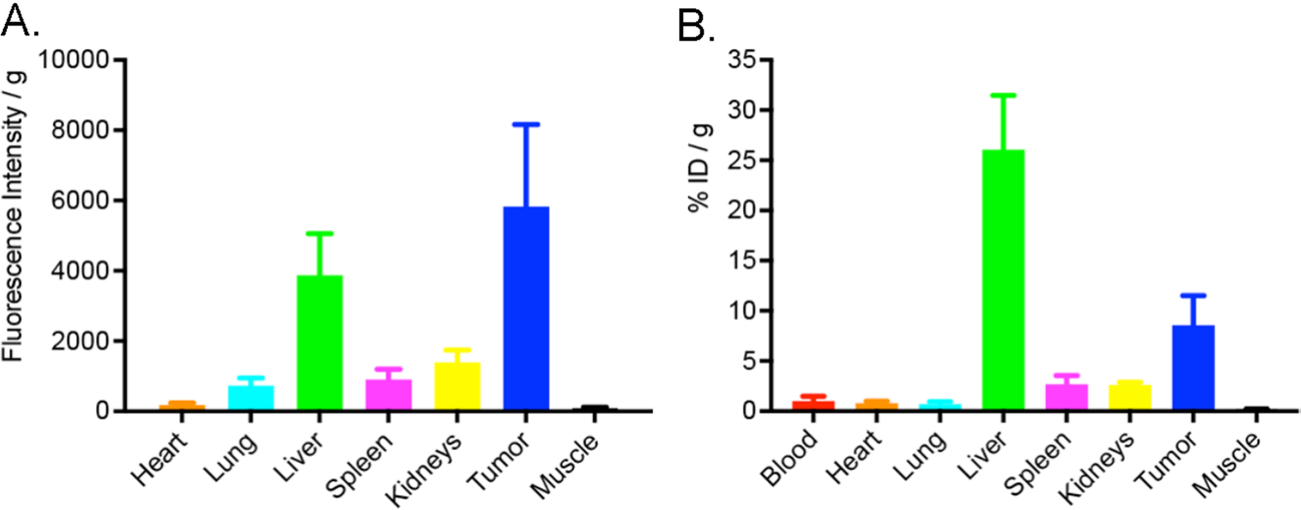
^89^Zr-DFO-M5A-SW-IR800 biodistribution. After PET and intra-vital optical imaging, the organs were excised, weighed, organ fluorescence intensity activity and radioactivity measured. The tissue biodistribution of fluorescence intensity per gram (**A**) and radioactivity % ID/g (**B**) were plotted with standard error of the mean

Post imaging, the organs were excised, weighed, organ fluorescence intensity activity and radioactivity measured. The biodistribution of fluorescence intensity per gram (standard error of the mean) and radioactivity percent injected dose per gram (%ID/g) are plotted in Fig. 4A. The liver was the major organ of uptake. While less than the tumor, the tumor/liver ratio (T/L) was 1.5. Liver clearance was observed over time, reflecting the same route of clearance as immunoglobulins [25]. There was low activity observed for the spleen, stomach, and kidney. The terminal radioactive biodistribution data expressed as percent injected dose per gram (% ID/g) confirmed a ~ 3 × fold higher signal in the liver compared to the tumor (Fig. 4B).

**Fig. 5:**
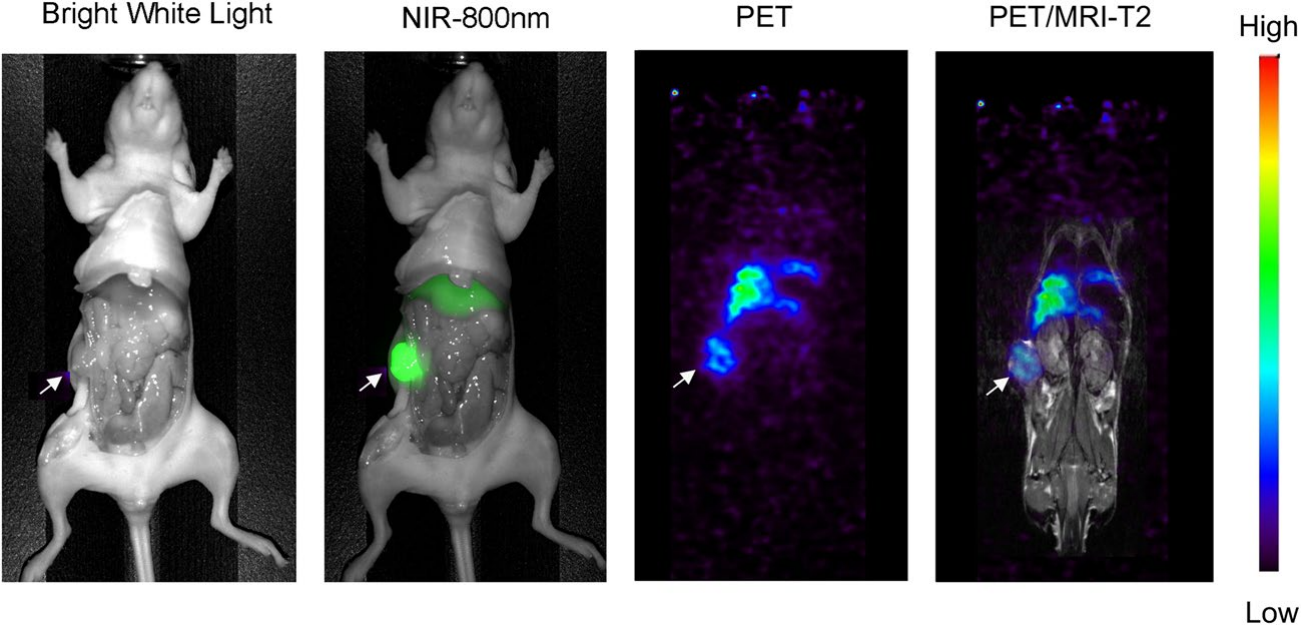
Multimodality imaging. Bright white light, NIR-800 fluorescence, immunoPET and PET/MR1 T2 scans demonstrate multimodality imaging of ^89^Zr-DFO-M5A-SW-IR800 in a representative mouse bearing a human colorectal cancer xenograft

Coincident with the PET imaging, we performed synchronous MR imaging using T2-weighted scans acquired for anatomical registration. For comparison of the multimodality imaging scans, a panel of bright white light, NIR-800 fluorescence, multiplanar reconstruction (MPR) immunoPET, and PET/MRI-T2 scans are displayed from a representative mouse (Fig. 5). The MPR/PET image is a better view for image comparisons, as it is a planar view rather than the maximum intensity projection (MIP) images (Fig. 2) that show total activity. The combined NIR/PET/MR imaging modalities showed excellent correlation for tumor, liver, and other anatomical features.

**Fig. 6:**
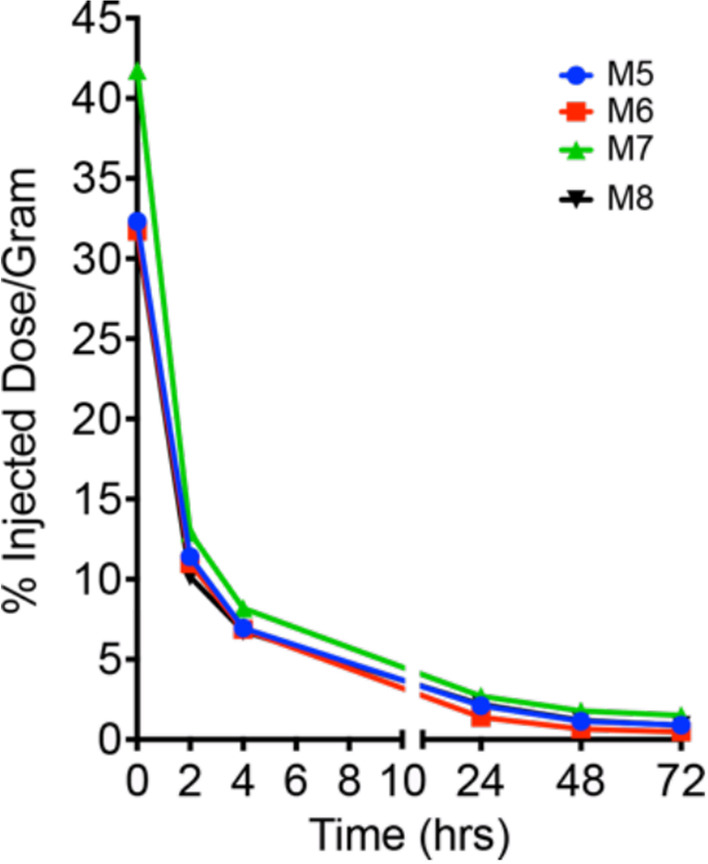
Blood clearance. ^89^Zr-DFO-M5A-SW-IR800 serial blood clearance in 4 mice bearing human colorectal cancer xenografts, reported as percent injected dose per gram

The 4 other mice injected with ^89^Zr-DFO-M5A-SW-IR800 were used for serial blood draws at 0, 2, 4, 24, 48, and 72 h. The volume was measured, radioactivity counted, corrected for radiodecay back to time of injection, allowing blood clearance data to be reported as a percent of the injected dose per gram (%ID/g) mean values with standard errors in Fig. 6. The blood clearance curves appears much faster than previously reported for the non-radiolabeled M5A-SW-IR800 [19].

## Discussion

The dual-labeled anti-CEA ^89^Zr-DFO-M5A-SW-IR800 antibody-dye conjugate was evaluated for NIR fluorescence and PET/MR imaging, terminal tissue biodistribution, and blood clearance in a human colorectal cancer LS174T xenograft mouse model. The NIR fluorescence imaging showed high specific tumor accumulation (Fig. 3). This was confirmed by the corresponding immunoPET scans. However, the Zr-89 PET MIP images showed much higher liver activity than the tumor (Fig. 2). To compare the biodistributions from the 2 orthogonal modalities, we compared their tumor to liver ratios. The NIR fluorescence had a T/L of 1.5 whereas counting radioactivity, the T/L was 0.33. Surprisingly, the NIR fluorescence imaging provided a better homogenous optical visualization of the tumor compared to the PET scan. The improvement in tumor visualization using optical imaging as compared to PET imaging was unexpected. The absolute quantity of the probe at the tumor and the liver is unchanged between the two imaging modalities, with the probe carrying an estimated 6 fluorophores and 4–5 chelates per antibody. The observed differences are likely driven by a combination of tracer and imaging system properties. The higher liver signal using radionuclide imaging as compared to the optical imaging may be due to the difference in the tissue depth of penetration of the two techniques. The tumor homogeneity of the NIR signal can be attributed to a higher resolution from the epifluorescence signal that has a higher signal to noise ratio. The faster blood clearance observed could also result in higher liver accumulation which is the site of antibody catabolism. This was not observed for the previous non-radioactive bromo-conjugate M5A-IR800-SW format [19], allowing speculation the DFO chelate’s charge/hydrophobicity may also be contributing to increased liver uptake and retention. These features highlight the advantages and disadvantages of the two imaging modalities. At the surface level, optical imaging has excellent sensitivity and spatial resolution, and PET imaging provides depth penetration and quantitation, providing complementary imaging but each effecting the physiological properties of the dual probe.

First in man studies utilizing antibody-based dual-modality probes are reaching the clinic and shown to be safe and useful for intraoperative guidance [26]. A combined dual modality probe offers advantages in the integration data from different imaging techniques, allowing for more accurate and comprehensive imaging [5]. The combination of both imaging modalities counteracts the poor spatial resolution of radionuclide imaging and the limited depth penetration of fluorescence optical imaging [15]. When the two complementary modalities are used intraoperatively in real time, radionuclide PET probes can facilitate identification of deep lesions such as lymph nodes or metastases. Widespread adoption of intraoperative radionuclide detection in sentinel lymph node surgery has led to the development of versatile and responsive platforms for detection in both open and laparoscopic surgery. Once localized and brought into the field, optical imaging provides detailed in-situ data to complement anatomic information, determine margins, and ensure completeness of surgery.

Drawbacks to a dual modality probe include the increased complexity in in synthesis of the molecule, the pharmacokinetics properties of each tracer affecting the final molecule, and a lack of flexibility when only one imaging modality is desired. This was encountered when determining the optimal dosage of anti-CEA ^89^Zr-DFO-M5A-SW-IR800 for our studies as the protein dose administered was higher than for just PET imaging to enable optimum optical imaging. The lack of flexibility in a dual-modality single agent probe can be an issue when only one imaging modality is desired, as the patient would receive a radionuclide agent when only optical imaging was necessary or if targeted-imaging needed to be performed for treatment planning. If a pre-surgical targeted scan was needed, a combined probe would limit time to interpretation and surgery as the agent is timed for optimal imaging at the same time. This would certainly be feasible in a sophisticated multidisciplinary cancer care setting where complex sequencing of imaging and treatment can be well-sequenced but may be limited in settings where such resources are unavailable. However, the potential complementary features of this probe make it ideal as a single agent for intraoperative surgical navigation. Enhanced efficiency in the development of a single administration of a dual modality agent can potentially reduce the of cost of technology development and clinical integration. A multi-modal probe such as the one described in this work utilizes the strengths of both imaging modalities to overcome their individual limitations, an attractive potential that supports development of such technology.

As we develop the ^89^Zr-DFO-M5A-SW-IR800 probe, we foresee the potential clinical utility of a dual-labeled probe to pre-operatively scan patients, perform fluorescence image guided surgery and/or radionuclide image guided surgery [27], back table histology analysis and to rescreen by PET for therapeutic efficacy with a single agent.

## Conclusion

In summary, the anti-CEA ^89^Zr-DFO-M5A-SW-IR800 shows the potential for high sensitivity and selectivity for NIR fluorescence/PET/MR multimodality imaging for intraoperative surgery. This work provides evidence toward translation as dual-labeled surgical oncology agent.

## Supplementary Information

Supplemental Scheme 1 Synthesis of Anti-CEA DFO-M5A-IR800 Sidewinder. The dPEG sidewinder NH_2_-dPEG_12_-Lys(t-boc)-NH-m-dPEG_24_ (Compound 1) was reacted with azido-PEG4-NHS to form Compound 2. The azido-SW-IR800 was obtained by removing the tert-butyloxycarbonyl (t-boc) protecting group and reacting with the non-aryl sulfonated IR800 nm dye to form Compound 3. (PNG 343 kb)

High resolution image (TIF 55 kb)

Supplemental Figure 2 Mass spectrometry analysis. The DFO-M5A-SW-IR800 antibody conjugate was reduced, and light and heavy chains analyzed by Agilent 6520 QTOF mass spectrometry. Masses were determined for DFO, SW-IR800, SW-IR800 +DFO and X unknown peak. (PNG 315 kb)

High resolution image (TIF 518 kb)

## Data Availability

Data that support the findings of this study are available from the corresponding author upon reasonable request.

## Abbreviations

PET: Positron emission tomography
FGS: Fluorescence guided surgery
DFO: Deferoxamine
^89^Zr: Zirconium-89
MRI: Magnetic resonance imaging
NIR: Near infrared
h: Hour
CEA: Carcinoembryonic antigen
CT: Computer tomography
dPEG: Defined polyethylene glycol
HPLC: High performance liquid chromatography
TCEP: Tris (2-carboxyethyl) phosphine
RT: Room temperature
SEC: Size exclusion chromatography
ATCC: American Type Cell Culture
FBS: Fetal bovine serum
DMEM: Dulbecco’s modified eagle’s medium
IVIG: Intravenous immune globulin
MIP: Maximum intensity projection
MPR: Multiplanar reconstruction
T2: Transverse relaxation time
