# Supplementary figures and images for: Multimodality PET and Near-Infrared Fluorescence Intraoperative Imaging of CEA-Positive Colorectal Cancer
Source: Mol Imaging Biol. 2023 Jun 21;25(4):727–34. doi: 10.1007/s11307-023-01831-8 (PMC10333401; doi:10.1007/s11307-023-01831-8)

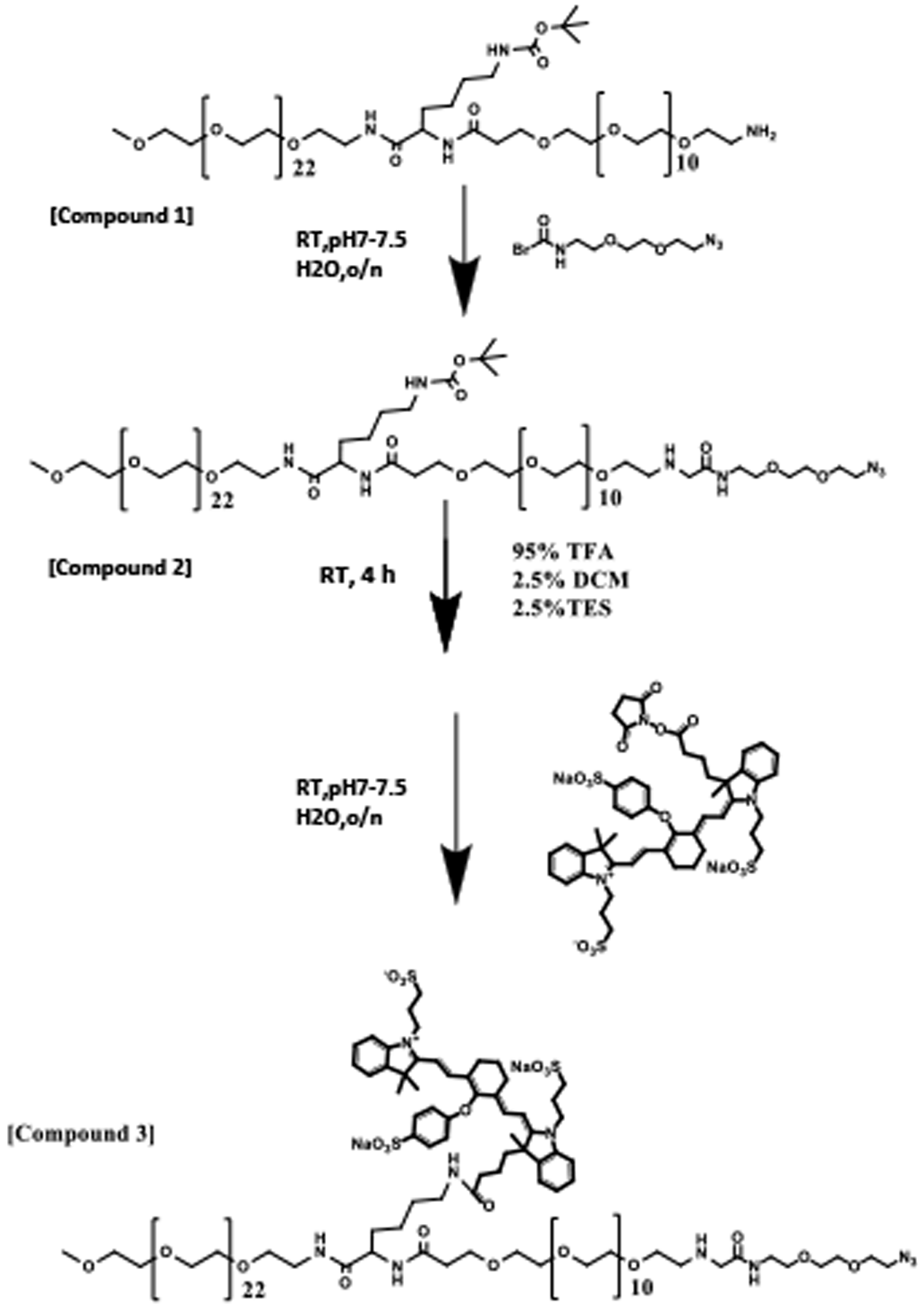

Supplement: Supplementary file 1 — Synthesis of Anti-CEA DFO-M5A-IR800 Sidewinder. The dPEG sidewinder NH2-dPEG12-Lys(t-boc)-NH-m-dPEG24 (Compound 1) was reacted with azido-PEG4-NHS to form Compound 2. The azido-SW-IR800 was obtained by removing the tert-butyloxycarbonyl (t-boc) protecting group and reacting with the non-aryl sulfonated IR800 nm dye to form Compound 3. (PNG 343 kb) [file 11307_2023_1831_Fig7_ESM.png]

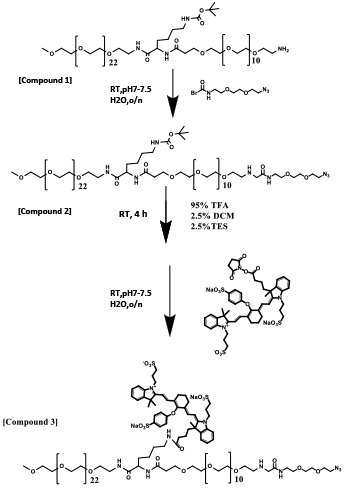

Supplement: Supplementary file 2 — High resolution image (TIF 55 kb) [file 11307_2023_1831_MOESM1_ESM.tif]

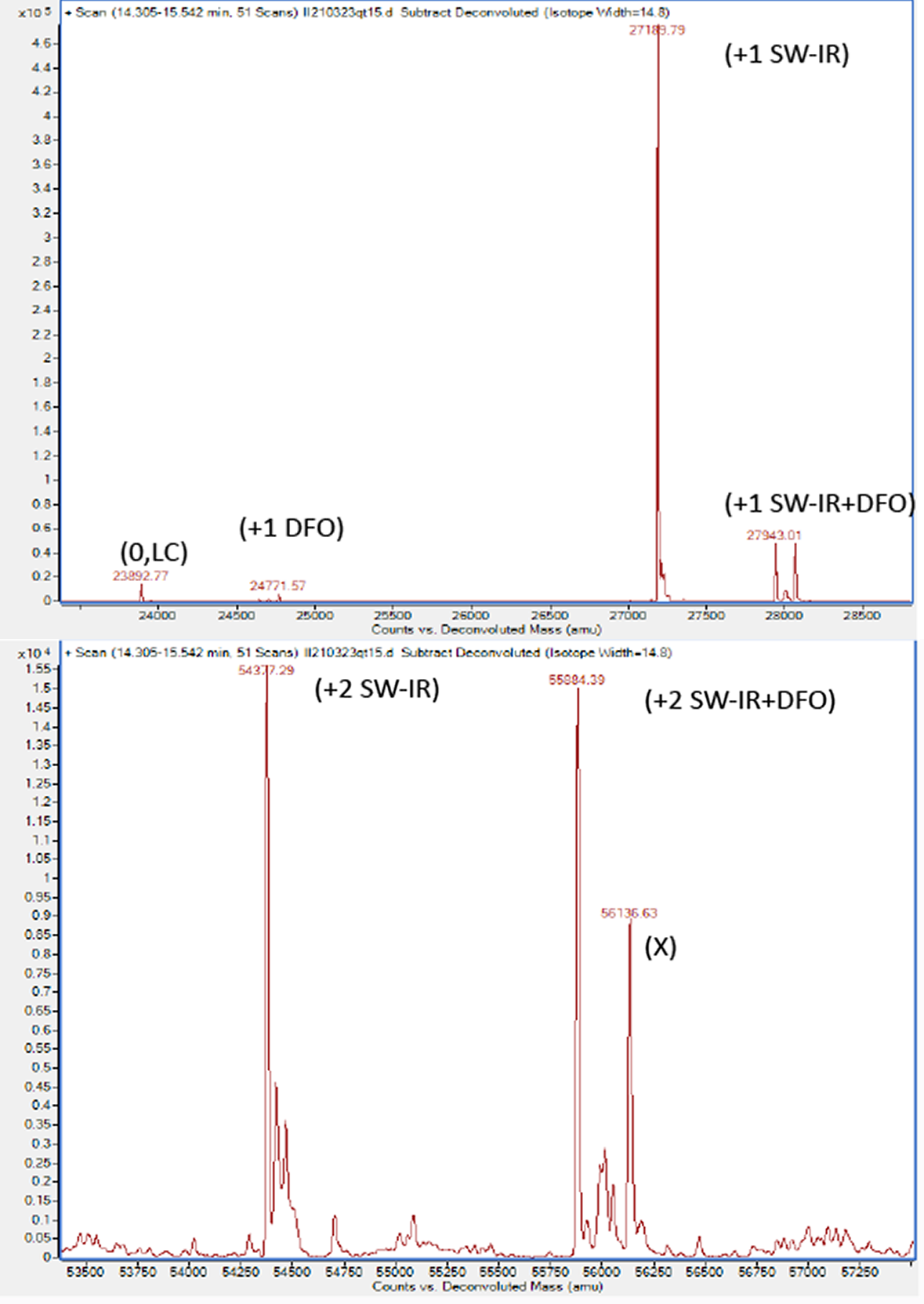

Supplement: Supplementary file 3 — Mass spectrometry analysis. The DFO-M5A-SW-IR800 antibody conjugate was reduced, and light and heavy chains analyzed by Agilent 6520 QTOF mass spectrometry. Masses were determined for DFO, SW-IR800, SW-IR800 +DFO and X unknown peak. (PNG 315 kb) [file 11307_2023_1831_Fig8_ESM.png]

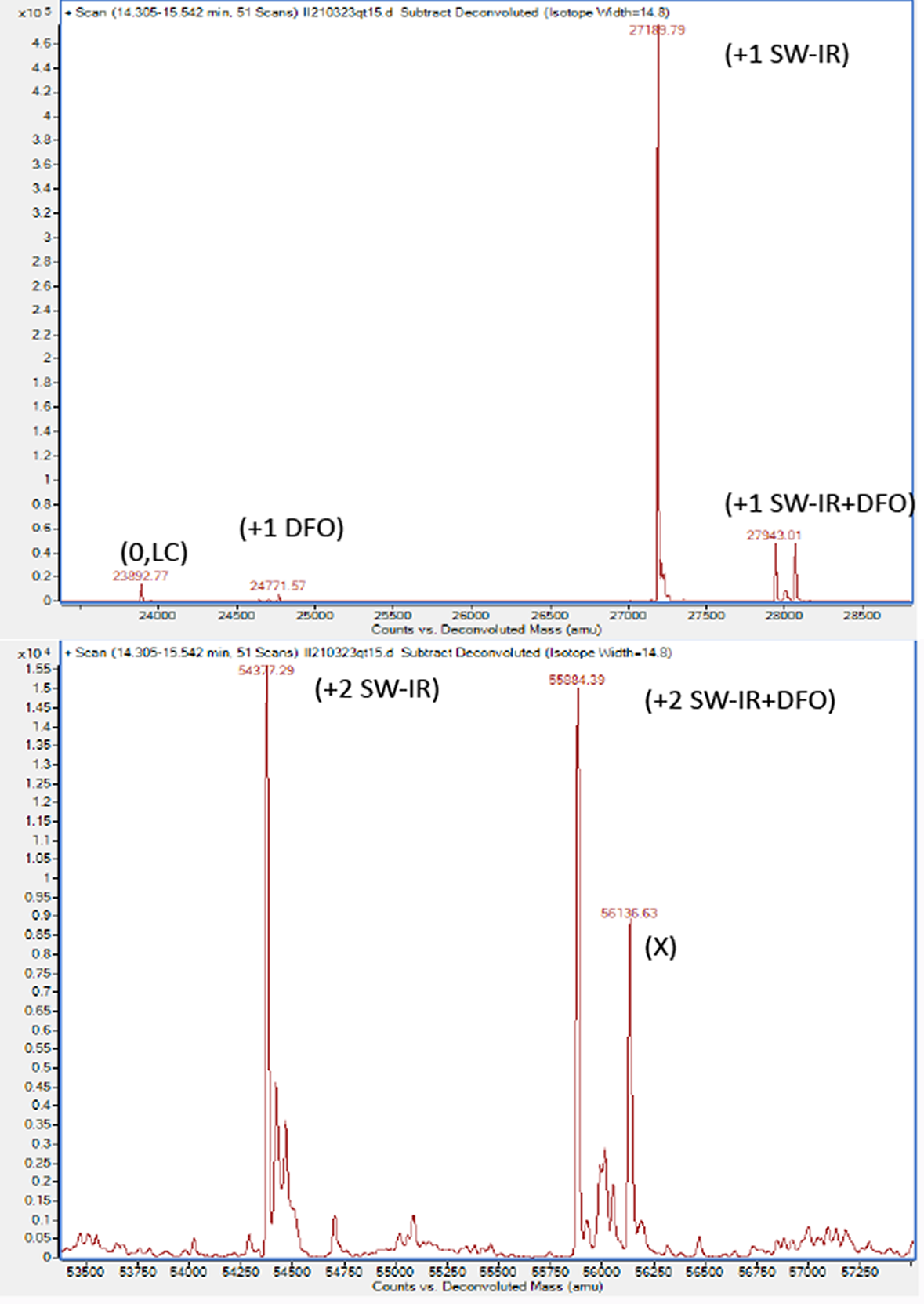

Supplement: Supplementary file 4 — High resolution image (TIF 518 kb) [file 11307_2023_1831_MOESM2_ESM.tif]
